# How to choose the most appropriate cognitive test to evaluate cognitive complaints in primary care

**DOI:** 10.1186/s12875-017-0675-4

**Published:** 2017-12-16

**Authors:** Jolien Janssen, Paula S. Koekkoek, Eric P. Moll van Charante, L. Jaap Kappelle, Geert Jan Biessels, Guy E. H. M. Rutten

**Affiliations:** 1 Julius Centre for Health Sciences and Primary Care, University Medical Centre Utrecht, Utrecht, The Netherlands; 2 Department of Neurology, Brain Centre Rudolf Magnus, University Medical Centre Utrecht, Utrecht, The Netherlands; 3 Department of General Practice, Academic Medical Centre, Amsterdam, The Netherlands

**Keywords:** Dementia, Primary care, Diagnostic tests, Aging, Alzheimer’s disease/dementia

## Abstract

**Background:**

Despite the wealth of research devoted to the performance of individual cognitive tests for diagnosing cognitive impairment (including mild cognitive impairment and dementia), it can be difficult for general practitioners to choose the most appropriate test for a patient with cognitive complaints in daily practice.

In this paper we present a diagnostic algorithm for the evaluation of cognitive complaints in primary care. The rationale behind this algorithm is that the likelihood of cognitive impairment -which can be determined after history taking and an informant interview- should determine which cognitive test is most suitable.

**Methods:**

We distinguished three likelihoods of cognitive impairment: not likely, possible or likely. We selected cognitive tests based on pre-defined required test features for each of these three situations and a review of the literature. We incorporated the cognitive tests in a practical diagnostic algorithm.

**Results:**

Based on the available literature, in patients with complaints but where cognitive impairment is considered to be unlikely the clock-drawing test can be used to rule out cognitive impairment. When cognitive impairment is possible the Montreal cognitive assessment can be used to rule out cognitive impairment or to make cognitive impairment more likely. When cognitive impairment is likely the Mini-Mental State Examination can be used to confirm the presence of cognitive impairment.

**Conclusions:**

We propose a diagnostic algorithm to increase the efficiency of ruling out or diagnosing cognitive impairment in primary care. Further study is needed to validate and evaluate this stepwise diagnostic algorithm.

## Background

In case of cognitive complaints expressed by the patient or a relative, or suspicion of cognitive impairment by the general practitioner (GP), it is important to evaluate cognitive symptoms with a reliable and efficient diagnostic procedure. Differentiating between subjective cognitive complaints and cognitive impairment, i.e. mild cognitive impairment (MCI) or early dementia, can be difficult [1]. Yet, history taking and the informant interview provide crucial information for the diagnostic procedure. The GP can complement this information with additional cognitive tests to reach more certainty about the presence or absence of cognitive impairment [2].

A wealth of research is devoted to the performance of individual cognitive tests. However, the literature gives limited consideration of and guidance on which and how cognitive tests should be used in the context of the sequential and probabilistic nature of the diagnostic procedure. Since the true value of a test is determined by the extent to which it provides information on top of the information that has already been gathered [3], the choice of the most appropriate cognitive test should be based on the estimated likelihood that the patient has cognitive impairment.

In this paper, we propose a stepwise diagnostic algorithm for the evaluation of cognitive complaints in primary care, taking into account both the GP’s assessment of the likelihood of cognitive impairment and properties of the test.

## Methods

To optimise the selection of cognitive tests we distinguished three likelihoods of objective cognitive impairment in patients with cognitive complaints, namely 1: cognitive impairment is not likely; 2: cognitive impairment is possible, but activities of daily living (ADL) appear to be preserved (i.e. MCI); and 3: cognitive impairment likely and ADL is affected (i.e. dementia).

First, the authors (including both neurologists and GPs experienced in diagnosing cognitive impairment) discussed the required test features for each of these situations. Secondly, we performed a literature search on cognitive tests used in primary care. We searched for English language articles listed on PubMed from January 2000 to January 2017. We used the search terms ‘dementia’ and ‘cognitive’ combined with ‘screening’, ‘assessment’, ‘instrument’, ‘tool’ and ‘measure’ combined with ‘primary care’. Due to the large and heterogeneous body of literature, we limited our selection to systematic reviews and meta-analyses. Third, we selected the most appropriate cognitive tests in relation to the GP’s assessment of the likelihood of cognitive impairment. At last, we incorporated the cognitive tests in a practical diagnostic algorithm and completed this algorithm using current guidelines and consensus documents to determine the key points that should be addressed in the first steps of the diagnostic procedure.

### Required test features

For all three likelihoods of cognitive impairment we identified the cognitive tests of which appropriate cut-off scores had been reported in at least two independent studies.

#### Cognitive impairment not likely

When a patient complains but the GP considers cognitive impairment to be not likely, the prior probability that this patient has cognitive impairment is low and the chance this patient has dementia will be even lower [4]. The main objective of a cognitive test in this situation is to rule out cognitive impairment, in particular MCI. A test should have a high negative predictive value (NPV) and should preferably be brief. A high positive predictive value (PPV) is less relevant if one aims to rule out a condition, as a low PPV can be amended by performing an additional test in case of a positive test result. For this situation, we only considered tests that have been studied for MCI.

#### Cognitive impairment possible

This is the most challenging diagnostic situation, the “grey zone”. When the GP considers cognitive impairment to be possible, but ADL appears to be preserved, the prior probability that the patient has MCI, or possibly even dementia is substantial [4]. The main objective of a cognitive test in this situation is to distinguish between presence or absence of cognitive impairment. A cognitive test in this situation should therefore be able to detect MCI and dementia in a population with a moderately high prevalence of cognitive impairment. We may assume that a test validated for MCI with an adequate NPV, will also detect dementia. Therefore, we considered tests that have been studied for MCI only, or MCI and dementia. We prioritised a high NPV above a high PPV to avoid false reassurance.

#### Cognitive impairment likely

When the GP considers the likelihood of cognitive impairment to be high and ADL appears to be affected, the prior probability that this patient has dementia is high [4]. A cognitive test in this situation should therefore be able to detect dementia in a population with a high prevalence of cognitive impairment. For this situation, we only considered tests that have been studied for dementia. The main objective of a test in this situation is to confirm that the patient has dementia; a test with a high PPV for dementia is therefore preferred.

## Results

We critically appraised ten systematic reviews and two meta-analyses [5–16]. Only one review, which is based on the comprehensive research report produced by Kaiser Permanente Research Affiliates Evidence-based Practice Center, provided sufficient details to assess the value of cognitive tests for our algorithm [17]. It includes a dual independent review of studies on brief (i.e. administered within 10 min or self-administered within 20 min) cognitive tests conducted in a primary care setting.

### Selecting cognitive tests

#### Cognitive impairment not likely

As shown in Table 1, both the clock-drawing test [18] and the Montreal cognitive assessment (MoCA) [19] have a high (≥ 89%) NPV and a moderate PPV (≤50%) in populations with relatively low prevalence rates of MCI (14–24%). Taking into account their comparable diagnostic accuracy, the short administration time of the clock-drawing test (1–3 min) relatively to the MoCA (10 min), we selected the clock-drawing test for our algorithm. The clock-drawing test assesses multiple aspects of cognitive functioning, in particular visuospatial and praxis abilities. In contrast, the MoCA contains multiple subtests that tap into different cognitive domains and can thus provide some more information on the actual nature of the cognitive impairment.

**Table 1:** Evidence summary [17] of cognitive tests for MCI (MCI versus normal cognition, dementia not included)

Test	Studies (n)	Test time (min)	Cut-off score	Study population, % MCI	Number of participants analysed	Sens (95% CI)	Spec (95% CI)	PPV (95% CI)	NPV (95% CI)
CDT	3	1–3	≤ 9	48	465	41 (34, 47)	83 (78, 88)	69 (60, 77)	60 (55, 65)
			≤ 9	15	3198	58 (54, 63)	57 (55, 59)	19 (17, 22)	89 (87, 90)
			≤ 9	14	428	69 (56, 81)	63 (58, 68)	23 (17, 30)	93 (89, 96)
MMSE	2	7–10	< 28	84	91	47 (36, 59)	73 (45, 92)	90 (76, 97)	22 (11, 35)
			< 28	44	524	45 (39, 52)	80 (75, 84)	64 (56, 71)	66 (60,70)
MoCA	2	10	< 26	24	152	100 (91, 100)	50 (41,59)	39 (29, 50)	100 (94, 100)
			< 26	20	99	80 (56, 94)	76 (65, 85)	46 (29, 63)	94 (85, 98)

Only the studies reporting a cut-off score that was studied more than once are depicted in the table. *Abbreviations*: *CI*=Confidence Interval, *MCI* Mild Cognitive Impairment, *NR* Not Reported, *Sens* Sensitivity, *Spec* Specificity, *NPV* Negative Predictive Value, *PPV* Positive Predictive Value, *AUC* Area Under the Curve. Abbreviations cognitive tests: *CDT* ClockDrawing Test, *MMSE* Mini Mental State Examination, *MoCA* Montreal Cognitive Assessment

#### Cognitive impairment possible

As shown in Tables 1 and 2, all tests that have been studied for MCI only, or cognitive impairment, have limited PPVs (≤71%), with the exception of study populations in which MCI or cognitive impairment is highly prevalent (≥50%). The MoCA has the most favorable NPV (≥ 94%) for both MCI and cognitive impairment overall and was therefore selected for our algorithm.

**Table 2:** Evidence summary [17] of cognitive tests for cognitive impairment (dementia and MCI versus normal cognition)

Test	Studies (n)	Test time (min)	Cut-off score	Study population, % dementia / % MCI	Number of participants analysed	Sens (95% CI)	Spec (95% CI)	PPV (95% CI)	NPV (95% CI)
MoCA	1	10	< 26	8/19	107	86 (67, 96)	76 (65, 85)	56 (40, 71)	94 (85, 98)
Mini-Cog	2	3–4	2/3	40/12	371	84 (79, 89)	88 (81, 93)	92 (87, 95)	77 (70, 83)
			2/3	3/39	630	39 (34, 45)	78 (73, 82)	57 (49, 64)	63 (59, 68)
MMSE	3	7–10	23/24	4/26	269	53 (43, 64)	92 (88, 95)	71 (59, 81)	85 (78, 89)
			23/24	9/47	160	77 (67, 85)	70 (58, 80)	77 (67, 85)	70 (56, 80)
			23/24	4/5	1115	72 (62, 81)	89 (65, 99)	39 (32, 47)	97 (96, 98)

Only the studies reporting a cut-off score that was studied more than once are depicted in the table. *Abbreviations*: *CI* Confidence Interval, *MCI* Mild Cognitive Impairment, *NR* Not Reported, *Sens* Sensitivity, *Spec* Specificity, *NPV* Negative Predictive Value, *PPV* Positive Predictive Value, *AUC* Area Under the Curve. Abbreviations cognitive tests: *MoCA* Montreal Cognitive Assessment, *MMSE* Mini Mental State Examination

#### Cognitive impairment likely

Table 3 demonstrates that the Mental Status Questionnaire, the Short Portable Mental Status Questionnaire and the Memory Impairment Screen were only investigated in study populations with a prevalence of dementia ≤18% and it is therefore unclear if they are suitable in a situation with a high prior probability of dementia. The Abbreviated Mental Test and the Mini-Cog were both studied twice, once in a population with a very low prevalence of dementia (3% and 4% respectively) and once in a population with a high prevalence of dementia (29% and 40% respectively). In populations with a high prevalence of dementia the PPV of both tests was 71%. The MMSE (cut-off <24) has a comparable PPV (73%) in a population with a dementia prevalence of 28%. The NPV of all tests - with the exception of the Abbreviated Mental Test - was above 90%. In conclusion, both the Mini-cog and the MMSE with a cut-off <24 have favourable test features for this situation. Since the MMSE [20] is most frequently studied and well known, we selected this test as most suitable for our algorithm.

**Table 3:** Evidence summary [17] of cognitive tests for dementia (dementia versus no dementia)

Test	Studies (n)	Test time (min)	Cut-off score	Study population, % dementia	Number of participants analysed	Sens (95% CI)	Spec (95% CI)	PPV (95% CI)	NPV (95% CI)
AMT	2		7/8	29	269	42 (31, 53)	93 (89, 96)	71 (56, 83)	80 (74, 85)
			7/8	4	358	92 (64, 100)	95 (93, 97)	43 (25, 63)	100 (98, 100)
Mini-Cog	2	3–4	2/3	40	371	97 (93, 99)	71 (65, 77)	71 (64, 77)	97 (93, 99)
			2/3	3	630	76 (54, 90)	73 (69, 76)	9 (5, 14)	99 (97,100)
MIS	3	4	4	10	483	80 (66, 90)	96 (94, 98)	70 (57, 82)	98 (96, 99)
			4	18	318	76 (42, 100)	73 (56, 96)	38 (29, 47)	94 (89, 97)
			4	12	240	86 (67, 96)	97 (94, 99)	80 (61, 92)	98 (95, 100)
MMSE	5	7–10	23/24	4	1115	91 (78, 98)	87 (85, 89)	23 (17, 29)	100 (99, 100)
			23/24	1	709	87 (78, 95)	89 (86, 92)	52 (44, 60)	98 (96, 99)
			23/24	4	358	77 (46, 95)	97 (94,98)	46 (24, 68)	99 (97, 100)
			23/24	6	648	88 (74, 96)	88 (85, 90)	32 (24, 42)	99 (98, 100)
			23/24	28	360	84 (75, 90)	88 (84, 92)	73 (64, 81)	94 (90, 96)
MMSE	4	7–10	24/25	29	283	81 (70, 88)	76 (70, 82)	57 (48, 67)	90 (85, 95)
			24/25	4	269	98 (78, 100)	84 (79, 87)	21 (13, 33)	100 (99, 100)
			24/25	14	274	85 (70, 94)	81 (75, 86)	42 (31, 54)	97 (94, 99)
			24/25	19	449	84 (75, 91)	83 (79, 87)	55 (46, 63)	96 (93, 98)
MSQ	2	4	7/8	16	164	100 (87, 100)	84 (76, 89)	54 (39, 69)	100 (97, 100)
			7/8	4	358	92 (64, 100)	98 (96, 99)	67 (41, 87)	100 (98, 100)
SPMSQ	2	3–4	7/8	3	119	100 (29, 100)	100 (97, 100)	100 (29, 100)	100 (97, 100)
			7/8	4	358	100 (75, 100)	97 (94, 98)	54 (33, 75)	100 (99, 100)

Only the studies reporting a cut-off score that was studied more than once are depicted in the table. *Abbreviations*: *CI* Confidence Interval, *MCI* Mild Cognitive Impairment, *NR* Not Reported; *Sens* Sensitivity, *Spec* Specificity, *NPV* Negative Predictive Value, *PPV* Positive Predictive Value, *AUC* Area Under the Curve. Abbreviations cognitive tests: *AMT* Abbreviated Mental Test, *MIS* Memory Impairment Screen, *MMSE* Mini Mental State Examination, *MSQ* Mental Status Questionnaire, *SPMSQ* Short Portable Mental Status Questionnaire

### Proposed algorithm for a cognitive evaluation

#### Cognitive complaints

The starting point of the algorithm (1.1) is cognitive complaints expressed by the patient or a relative, or suspicion of cognitive impairment by the GP. In the evaluation of cognitive complaints, the mode of onset (1.2) provides essential guidance. In MCI and dementia, which is mostly caused by neurodegenerative or vascular pathologies, cognitive impairment is acquired and has a slowly progressive onset. This algorithm is not applicable to cognitive symptoms that develop within days or weeks. In that situation, other diagnoses such as a delirium or other neurological conditions are more likely.

#### History taking and informant interview

History taking and an informant interview are fundamental in a cognitive evaluation [2]. Concerns expressed by a close informant are generally even more predictive of cognitive impairment than self-reported symptoms [2]. An informant interview is preferably performed with a close informant separately from the patient. If an informant is not available and diagnostic uncertainty persists after the initial visit, the patient should bring an informant to a follow-up visit. The following topics should be addressed:

##### Nature and course of the symptoms

The GP should ascertain when and how symptoms started and how these developed over time. Memory problems are typically one of the first symptoms of cognitive impairment, but other cognitive domains may also be affected (Table 4) [21].

**Table 4:** Signs and symptoms to discuss during history taking and to help signalling cognitive impairment [21]

Memory impairment	
• Repeating questions or conversations • Hesitations, inconsistencies, omissions or confabulations • Head turning sign (to verify answers with a caregiver) • Misplacing personal belongings • Forgetting events or appointments • Getting lost on a familiar route	
Aphasia	
• Difficulty thinking of common words while speaking or using incorrect words • No fluent production of words	
Apraxia	
• Difficulties in performing or imitating simple tasks (such as combing hair or brushing teeth) with intact comprehension, motor skills and perception	
Agnosia	
• Impaired ability to recognise faces or common objects or to find objects in direct view despite good acuity (visual agnosia) • Impaired ability to recognise or identify objects by touch alone (tactile agnosia)	
Disturbance in executive functioning	
• Not correcting mistakes • Difficulty learning how to use a new gadget or machine around the house • Inability to manage finances • Loss of abstract thinking, logical reasoning and/or visuoconstruction (e.g. drawing a clock) • Lack of insight in own functioning • Loss of initiative, increased impulsivity or uninhibited behaviour	

##### Personality and behavioural changes

Changes in personality and behaviour are common in people with cognitive impairment and can cause considerable distress for both the patient and relatives. The Neuropsychiatric Inventory Questionnaire is frequently recommended to assess severity and impact of behavioural changes [22, 23]. The score of this 12-item informant questionnaire ranges from 0 to 36 with higher scores indicating more behavioural disturbance [24].

##### Depressive symptoms

GPs should be alert for depressive symptoms in patients with suspected cognitive impairment [25]. Depression can be a prodromal symptom of dementia but depressive symptoms can also follow cognitive decline. In addition, depressive symptoms can influence cognitive testing. If a depression is likely, focus should be on diagnosing and treating depression first. For his, a depression scale, such as the 15-item Geriatric Depression Scale, can be used [26]. Cognitive symptoms should always be re-evaluated after the depression is treated.

##### Risk factors

Age is the most important predisposing risk factor for cognitive impairment with estimated prevalence rates around 1% at the age of 60 and 30–60% in individuals of ≥90 years [27]. Lower intelligence, education and occupational attainment are associated with a higher risk of developing cognitive impairment [28]. Additional risk factors are a positive family history (especially early-onset cases) and head trauma [29]. Diabetes and cardiovascular risk factors, such as smoking and hypertension are other predisposing factors [30].

##### Daily functioning

Daily functioning comprises ADL and instrumental ADL (IADL). ADLs are basic daily self-care activities including feeding, bathing, dressing, mobility, toileting and continence. IADLs are more advanced activities including telephone use, shopping, food preparation, housekeeping, laundry, transportation, responsibility for medication and handling finances. In patients with MCI, ADLs are preserved while there can be minimal impairment in IADLs [31]. In patients with dementia (I)ADLs are affected by definition [32]. It should be noticed that the boundaries between “normal” and “impaired” daily functioning are not always evident and are influenced by pre-existent activity levels. The Katz ADL [33] and the Lawton IADL [34] scales are frequently recommended to assess (I)ADL. Both scales can be completed by the patient or an informant.

#### Is cognitive impairment not likely, possible or likely?

**Fig. 1:**
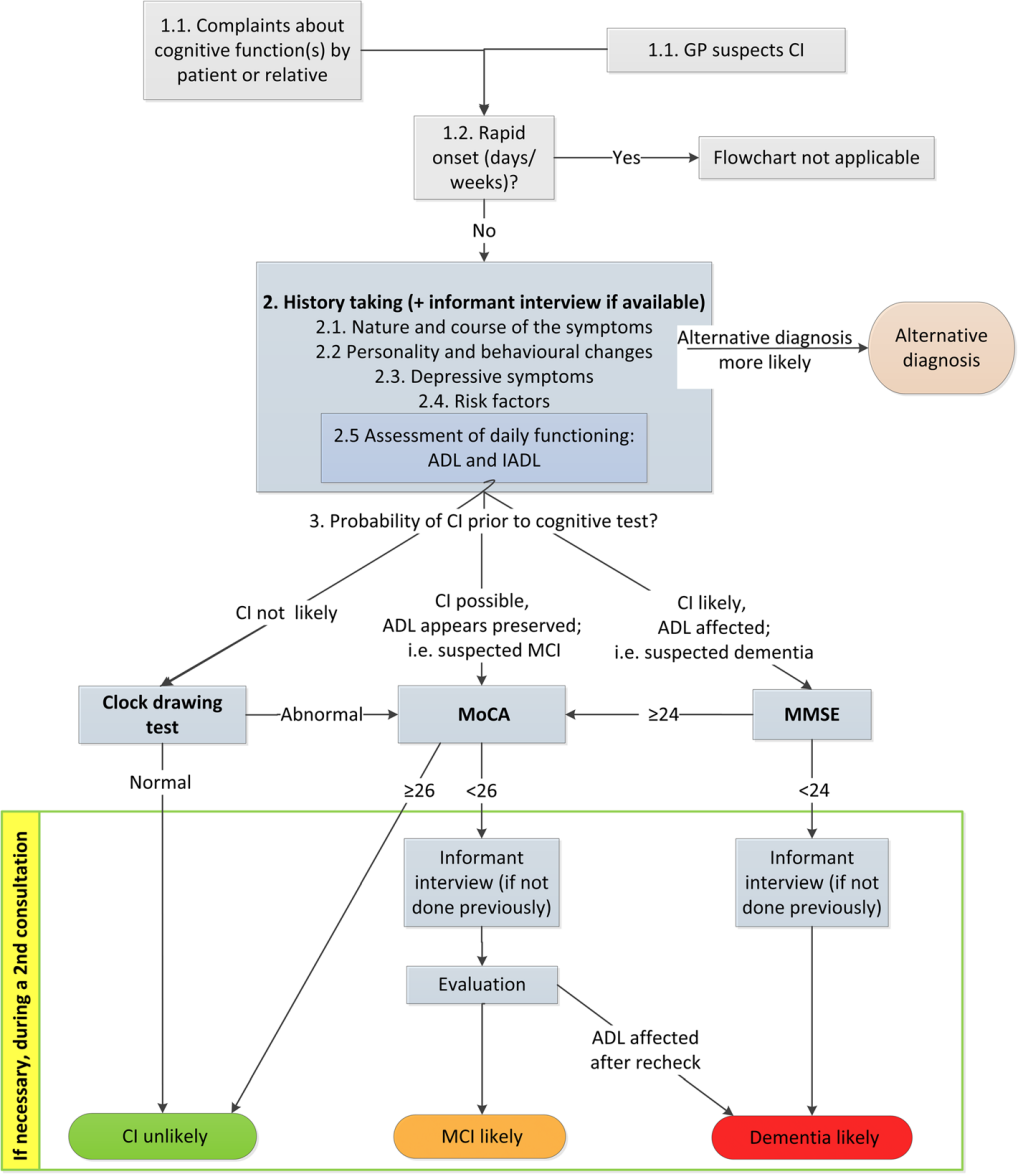
Diagnostic algorithm for the evaluation of cognitive complaints in primary care

Based on the previous steps the GP can estimate the likelihood that the patient has cognitive impairment and choose the most suitable cognitive test (Fig. 1). If according to the GP the likelihood that the patients has cognitive impairment is very low or very high, it may well be that none of the cognitive tests are of added value. Not using any cognitive test could then be a good option.

#### What if the cognitive test result does not match the GPs expectations?

The steps in the proposed algorithm will guide the GP towards the most probable diagnosis (Fig. 1). However, if there is a mismatch between the findings of history taking and the test, the results need to be reconsidered. It is important to perform an informant interview if not done previously and to consider alternative diagnoses. If uncertainty persists, the GP may decide to re-evaluate the patient in 6–12 months or to refer to a specialist for a more comprehensive cognitive assessment.

## Discussion

Current guidelines and guidance articles about which, when and how to use cognitive test during a cognitive evaluation in primary care are diverse. Most often the same cognitive test(s) are recommended for *all* patients who consult the GP with cognitive complaints regardless of the prior probability of cognitive impairment [21–23, 35–41]. The MMSE is most frequently recommended, followed by the MoCA, the clock-drawing test and the Mini-Cog. The choices of the cognitive tests in our algorithm are therefore consistent with current recommendations. However, we recommend the use of three different tests in three different situations to make the diagnostic procedure more efficient and tailored to the individual patient.

To our knowledge, this is the first time that a diagnostic algorithm is presented where the choice of cognitive tests is guided by the prior probability that the patient has cognitive impairment. This allows the GP to take into account the true value of a test, in addition to information that has already been gathered. For example, the short and sensitive clock-drawing test will have no added value in patients who visit the GP with typical signs and symptoms of dementia and the added value of a normal MMSE score is limited in patients with only mild symptoms of cognitive impairment. It can therefore be expected that our algorithm is more efficient, although its true value should still be established.

Several limitations of our approach in constructing the algorithm should be considered. The information on test characteristics of many tests was limited. Only a few tests have been studied in more than one fair or good quality study that included specific cut-off values. Hence, at present the available evidence to select suitable cognitive tests for the diagnostic algorithm was limited. Prioritising test characteristics is to a certain extent subjective, we tried to avoid subjectivity as much as possible by means of pre-defined criteria based on expert opinion and consensus; however, other opinions are possible and could lead to the selection of other cognitive tests. In addition, we had to make assumptions about the pre-test probability in each of the three situations we distinguished. Further study is needed to validate and evaluate this diagnostic algorithm.

## Conclusions

In conclusion, the ‘one-size-fits-all’ approach for patients with cognitive complaints appears obsolete. The prior probability that the patient has cognitive impairment should be taken into account when choosing a cognitive test. The algorithm reflected in Fig. 1 may guide GPs during this diagnostic procedure.

## Abbreviations

ADL: Activities of daily living
CI: Cognitive impairment (includes MCI and dementia)
GP: General practitioner
IADL: Instrumental activities of daily living
MCI: Mild cognitive impairment
MMSE: Mini mental state examination
MoCA: Montreal cognitive assessment
NPV: Negative predictive value
PPV: Positive predictive value
